# Significant costs and low utilization of stored peripheral blood stem cells for salvage autologous transplant in multiple myeloma patients including those meeting mSMART criteria

**DOI:** 10.1038/s41409-021-01223-y

**Published:** 2021-01-29

**Authors:** Nausheen Ahmed, Lucy Li, Patricio Rojas, Fahrettin Covut, Jane Reese-Koc, Merle Kolk, Ehsan Malek, Leland Metheny, Timothy O’Brien, Paolo Caimi, Marcos de Lima, Brenda W. Cooper

**Affiliations:** 1 Seidman Cancer Center, University Hospitals, Cleveland Medical Center, Case Western Reserve University, Cleveland, OH USA; 2 Case Western Reserve University, Cleveland, OH USA

**Keywords:** Stem-cell research, Disease-free survival, Myeloma

## To the Editor:

Autologous stem cell transplantation (ASCT1) plays an important role in the management of patients with multiple myeloma (MM) and remains relevant in the era of novel therapeutics [1, 2].

A second autologous transplant, usually after reinduction therapy, is a recommended strategy at the time of relapse, with improved progression-free survival (PFS) and possibly improved overall survival (OS) in select patients compared to chemotherapy in some series [3–5]. Due to potential difficulties harvesting adequate peripheral blood stem cells (PBSC) at the time of relapse and published clinical guidelines our institutional policy has been to collect and cryopreserve sufficient hematopoietic progenitor cells whenever feasible to allow for salvage transplant (ASCT2) at the time of relapse. The utilization of salvage autologous transplants and associated costs were analyzed in a cohort of 169 MM patients who received ASCT1 between January 2009 and December 2017. We focus, in particular on the subgroup of patients who met the mSMART criteria for ASCT2 defined as relapse >36 months with the use of maintenance therapy or 18 months without the use of maintenance therapy, respectively [6].

Patient demographic data, disease-related information, and cellular therapy data were obtained from institutional databases, the Stem Soft Database and chart review. Follow-up was until death or last contact with the data cutoff being January 1, 2020. Patients were censored at their last visit if they were lost to follow-up. This study was approved by the Institutional Review Board at University Hospitals, Cleveland Medical Center, and due to the nature of the study, patient consent was not required. ASCT2 was offered based on patient and physician preference.

PBSC were mobilized using cyclophosphamide (2–4 gm/m^2^) plus filgrastim (*n* = 24), filgrastim at a dose of 10–16 µgm/kg with leukapheresis starting on day 5 (*n* = 19), or filgrastim plus plerixafor (*n* = 123) and was not available for three patients. The minimum acceptable CD34 target collection goal was 5 × 10^6^ CD34+ cells per kilogram of actual body weight with extra days of collection defined as one or more additional days of leukapheresis for the sole purpose of obtaining ≥2 × 10^6^ CD34 cells/kg for storage. No additional collection charges were tabulated for patients who were unable to store adequate progenitor cells (i.e., <2 × 10^6^ PBSC/kg) or for patients who achieved their target goal in one pheresis session.

The median age of the 169 patients was 61 years (36–77 years) and the median time from diagnosis to first autograft was 9 months (range 3–115) and was more than 2 years in 14 patients (8.2%). Overall, 100 patients (59%) received one treatment regimen, 46 (27%) received two regimens and 23 (14%) received three or more regimens prior to autograft and 95% of patients had a clinical response prior to undergoing ASCT1. Adequate PBSCs for storage was achieved in a single leukapheresis for 30.2% of patients who received plerixafor and filgrastim compared to less than 10% using chemotherapy plus filgrastim or filgrastim alone (*χ*^2^ = 21.9, *p* < 0.05, Table 1a). The range of PBPC mobilized was 2.25–82.32 × 10^6^ CD34+ cells/kg (median 9.32 × 10^6^) and 18 and 5 patients, respectively, failed to mobilize adequate stem cells or had insufficient information, leaving a total of 146 patients available for analysis.

**Table 1:** Apheresis procedures and costs. (a) Apheresis procedures performed according to mobilization strategy (*n* = 169 patients). (b) Associated costs for stem cell collection and storage (*n* = 146).

(a)					
Total days	Chemotherapy plus filgrastim *n* (%)	Filgrastim alone *n* (%)	Plerixafor plus filgrastim *n* (%)	Unknown *n* (%)	Total *n* (%)
1	2 (8.3)	2 (10.6)	36 (29.3)	–	40 (23.7)
2	17 (70.9)	9 (47.4)	42 (34.2)	2 (66)	70 (41.4)
3	2 (8.3)	7 (36.8)	26 (21.1)	1 (33)	36 (21.3)
>4	3 (12.5)	1 (5.2)	15 (12.2)	–	19 (11.2)
Unknown	–	–	4 (3.2)	–	4 (2.4)
Total	24	19	123	3	169 (100)

(b)				
Procedure	Cost per procedure	Total extra procedures	Average additional cost per patient^a^	Total cost over 9 years
Leukapheresis	$ 6189/day	112 extra days of collection	$ 4748	**$ 693,168**
Cell processing and analysis	$ 11,009/day	112 extra days of collection	$ 8445	$1,233,008
Plerixafor use	$ 5622/dose	78 extra doses	$ 3003	**$ 438,516**
Storage	$ 5/month	Total 11,495 months	$ 393	$ 57,475
Cumulative costs	–	–	**$ 16,590**	**$ 2,422,167**

^a^Costs were calculated for 146 patients according to 2019 institutional charge master.

(a) Total days of collection for 169 patients according to mobilization strategy.

(b) Cost of extra collection for stem cell storage according to 2019 Institutional charge master.

At the time of data cutoff, only 3/146 (2%) patients with adequate stored PBSC received a salvage transplant. Overall, 129 patients (median follow-up 41 months) had complete follow-up information. Median PFS was 29 months among the 17 patients who did not receive maintenance therapy and 46 months in the 112 patients who received maintenance therapy (*p* = 0.11). At the time of this publication, 11/17 patients who did not receive maintenance therapy and 66/112 patients who received maintenance therapy relapsed, of whom 5 and 23 (total 28 patients) met mSMART criteria. Only three patients underwent ASCT2, (one of whom with relapse at 30 months did not meet mSMART criteria), experienced disease progression within 5–18 months and survived 25, 25, and 49 months after ASCT2. In comparison, median time to next treatment for the non-transplant cohort was 19 months, median survival was 45 months, and 12 patients were alive at data cutoff.

**Fig. 1:**
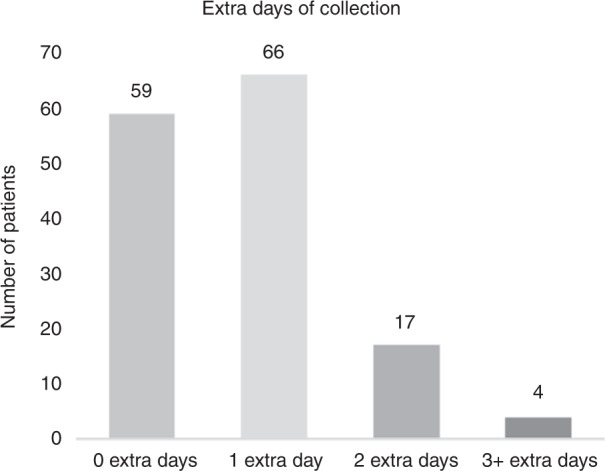
Extra days of collection to obtain adequate cells for storage. Extra days of collection required for patients to collect ≥2 × 106 CD34+ cells/kg for storage. The total number of extra days of collection was 112 days. 146 patients were included in this sample. 23 patients were excluded from the analysis due to failure to mobilize enough stem cells for storage (*n* = 18), or missing cell storage data (*n* = 5).

Figure 1 shows the distribution of 112 additional leukapheresis procedures in the 146 patients who had adequate cells stored for a second transplant. Costs associated with these extra collections tabulated for the entire cohort and per patient are shown in Table 1b.

We followed a large cohort of patients who received ASCT1 at our institution between 2009 and 2017 and with a median follow-up of 41 months, we found that only 3/146 patients (2%) who had available stored PBSCs underwent a salvage ASCT, leaving 98% of collected products unused. When we focused our analysis among the patients who met the more stringent mSMART criteria, only 3/28 patients (including 1 of who relapsed at 30 months) actually received ASCT2, leaving 90% of products unused in this more select group. Furthermore, among the 26 patients who met the mSMART criteria, sequential treatment with available novel therapeutics after relapse appeared to have non-inferior outcomes to ASCT2 in terms of time to next treatment and OS at 19 months and 45 months, respectively.

Due to the retrospective nature of our study, patient and physician factors that determined the selection of salvage strategy including quality of life concerns [7], could not be determined. Our data, however, are consistent with the utilization of salvage transplants worldwide which show a steady decline in the use of salvage ASCT [8, 9] This may be in part, due to the availability of novel salvage strategies as well as limited benefit, particularly after the failure of maintenance therapy [10].

The additional cost of collecting and storing these products totaled more than 2 million dollars, with an average additional cost per patient of US $16,590. Our costs are in line with those recently reported by others [9, 11]. Although the costs of obtaining and storing additional PBSCs is modest in the context of other medical treatments for these patients, this practice may add unnecessary cost burden to individual institutions.

At the time of this report about half of the patients continue to be free from relapse and 22 patients are within 3 years of ASCT1 and still have the option to use their cryopreserved products. In our study, none of the patients received stored stem cell products to “refresh” the bone marrow to allow further treatments in the setting of poor marrow reserve, which is another potential use of these products.

Given the limited use of salvage transplantation, the universal practice of collecting adequate PBSC for an initial and salvage transplant should be re-evaluated. Noting difficulties of remobilization after autologous transplantation ideally, a subset of patients could be selected based on pretransplant clinical and laboratory parameters, such as minimal residual disease testing and planned maintenance strategies to determine the population of patients who would be more likely to utilize ASCT2 at the time of relapse [12].
